# Investigating two decades of Streptococcus pneumoniae bacteraemia in the Gelderland area, the Netherlands, using whole-genome sequencing

**DOI:** 10.1099/mgen.0.001377

**Published:** 2025-03-18

**Authors:** Ana D. Sanches Ferreira, Alannah C. King, Femke Wolters, Heiman F.L. Wertheim, Bert Mulder, Caroline M.A. Swanink, Christa E. van der Gaast-de Jongh, Daan W. Arends, Nina M. van Sorge, Carel Schaars, Harry C. H. Hung, Paulina A. Hawkins, Lesley McGee, Stephen D. Bentley, Jan-Willem Veening, Marien I. de Jonge, Stephanie W. Lo, Amelieke J.H. Cremers

**Affiliations:** 1 Parasites and Microbes, Wellcome Sanger Institute, Hinxton, UK; 2 Department of Medical Microbiology, Radboudumc Center for Infectious Diseases, Nijmegen, The Netherlands; 3 Department of Pulmonology, Catharina Hospital, Eindhoven, The Netherlands; 4 Dicoon Laboratory, Elst & Department of Clinical Microbiology, Canisius-Wilhelmina Ziekenhuis, Nijmegen, The Netherlands; 5 Department of Clinical Microbiology and Immunology, Rijnstate, Arnhem, The Netherlands; 6 Department of Laboratory Medicine, Laboratory of Medical Immunology, Radboudumc Community for Infectious Diseases, Nijmegen, The Netherlands; 7 Netherlands Reference Laboratory for Bacterial Meningitis, Department of Medical Microbiology and Infection Prevention, Amsterdam University Medical Center, Amsterdam, The Netherlands; 8 Department of Internal Medicine, Pantein Maasziekenhuis, Boxmeer, The Netherlands; 9 Division of Bacterial Diseases, Centers for Disease Control and Prevention, Atlanta, USA; 10 Department of Fundamental Microbiology, Faculty of Biology and Medicine, University of Lausanne, Lausanne, Switzerland; 11 Milner Centre for Evolution, Department of Life Sciences, University of Bath, Bath, UK; 12 The Great Ormond Street Institute of Child Health, University College London, London, UK

**Keywords:** antimicrobial resistance, invasive pneumococcal disease (IPD), pneumococcal conjugate vaccine (PCV), pneumococcal lineage, serotype, the Netherlands

## Abstract

In the Netherlands, the 7-valent pneumococcal conjugate vaccine (PCV) was introduced to the childhood immunization programme in 2006 and replaced by the 10-valent PCV (PCV10, GSK) in 2011. To describe invasive pneumococcal disease in the era of childhood PCV vaccination on pneumococcal bacteraemia across all ages, we collected and sequenced 979 pneumococcal blood isolates from consecutive patients with pneumococcal bacteraemia in the Gelderland area, the Netherlands, between 2000 and 2020. In total, 58% of the bacteraemia cases (*n*=563/979) occurred in the elderly population. Compared to the pre-PCV period (2000–2005), the odds ratio for non-PCV10 bacteraemia was 17.5 (CI 9.9–31.6; *P*<0.001) in the late-PCV10 period, showing an overall increase in the proportion of bacteraemia cases being caused by non-vaccine serotype pneumococci (2016–2020). The increase in non-PCV10 serotypes is mainly driven by an expansion of lineage global pneumococcal sequencing cluster 3 (GPSC3) expressing serotype 8, alongside the emergence of serotype 12F that was mediated by multiple lineages (GPSC32/GPSC26/GPSC55). Both serotypes 8 and 12F were included in the latest PCV20 formulation that is licensed to be used in children and adults in Europe. Over 20 years, we observed a low prevalence of antimicrobial resistance (AMR) as predicted by genome data. There were no significant changes in AMR prevalence after vaccine introduction (*P*>0.05 for all comparisons). We saw a stably low prevalence of reduced penicillin susceptibility, which was observed in multiple pneumococcal lineages, with GPSC10 being the most common in the Gelderland collection, whilst GPSC1 and GPSC6 were common among the penicillin-resistant pneumococcal blood culture isolates provided by the Netherlands Reference Laboratory for Bacterial Meningitis. Comparison to global collections of GPSC10, GPSC1 and GPSC6 isolates favored the likelihood of separate introductions of penicillin-resistant isolates rather than cloncal expansion. Genomic surveillance of pneumococcal bacteraemia in this unbiased population sample in the Netherlands supports the use of higher valency PCVs, such as PCV20, especially in adults, to prevent future bacteraemia cases caused by *Streptococcus pneumoniae* in the Gelderland area, the Netherlands, while maintaining a low prevalence of AMR in the pneumococcal population.

**Impact Statement**

A 20-year dataset from the Gelderland area, the Netherlands, encompassing the timeframe of PCV7 and PCV10 introductions in the childhood vaccination programme, allowed for the identification of lineages and serotypes frequently seen amongst patients with bacteraemia. Cases of bacteraemia caused by non-PCV10 (GSK) serotypes increased over the time period. GPSC3 serotype 8 *Streptococcus pneumoniae* emerged as the main agent of bacteraemia after the introduction of PCVs, particularly in the adult and elderly age groups, along with GPSC26 serotype 12F. These lineages have previously been observed to rise also in other countries post-PCV13 introduction, suggesting that increases in GPSC3 and GPSC26 may be common more widely post-PCV introduction. Finally, this study provides findings to support the introduction of a PCV that includes serotypes 12F and 8 to extend serotype coverage to address the serotypes now causing bacteraemia among adult populations in the Gelderland area, the Netherlands.

## Data Summary

Genome sequences are available in the European Nucleotide Archive, the accession number, and a phylogenetic interactive visualization is available at https://microreact.org/project/gps2-netherlands. Metadata of the pneumococcal isolates included in this study is submitted as supplementary data and available on the GPS database https://data-viewer.monocle.sanger.ac.uk/project/gps. Supporting data, code and protocols provided as supplementary data files have been approved by all the authors.

## Introduction

*Streptococcus pneumoniae* (*pneumococcus*) is an opportunistic human pathogen that can cause mild infections such as otitis media, yet also severe infections such as bacteraemia, pneumonia and meningitis [1]. Invasive pneumococcal disease (IPD) cases are defined by the presence of *S. pneumoniae* in a normally sterile body site; it is most frequently detected by a positive blood culture with *S. pneumoniae*, affirming the presence of viable bacteria in the bloodstream (i.e. bacteraemia). Children and the elderly are at the greatest risk of bacteraemic pneumococcal disease and constitute the majority of cases [2]. Vaccination contributes to the prevention of pneumococcal disease and antibiotics to its treatment [3].

Globally, the introduction of pneumococcal conjugate vaccines (PCVs) has significantly reduced the incidence of IPD caused by serotypes targeted by the PCV [4,7]. However, a concurrent rise in IPD cases due to non-PCV serotypes has been observed. Countries that routinely used 10-valent PCV (PCV10, GSK) usually saw an increase in serotype 19A [8,10]. The distribution of emerging non-PCV13 serotypes varies geographically: serotypes 8, 12F and 24F are predominant in Europe [11,13]; serotypes 35B, 8 and 12F in Africa [4,14, 15]; serotypes 35B, 33F, 22F and 15A in North America [16]; and serotypes 6C, 12F and 24F in Latin America [5,8, 17]. Notably, serotypes 3, 19F and 19A have continued to cause disease despite the widespread use of PCV13 [14,18, 19]. The latest pneumococcal vaccine, PCV20, includes seven additional serotypes (8, 10A, 11A, 12F, 15B, 22F and 33F) compared to PCV13 and is anticipated to provide broader protection against emerging serotypes, although serotypes 24F and 35B remain outside its coverage.

In the Netherlands, the 7-valent PCV (PCV7) was introduced to the national paediatric immunization programme for all infants born after 1 June 2006 [20], and in May 2011 it was replaced by the PCV10 (GSK) [21]. There were no catch-up campaigns [21]. These vaccines target 7 and 10 pneumococcal serotypes, respectively, out of 106 known pneumococcal serotypes (https://www.pneumogen.net/gps/#/resources#serotypes, last accessed on 1 October 2024), with serotypes being defined by the serological recognition of the pneumococcal polysaccharide capsule (i.e. Quellung reaction). PCV7 includes the serotypes 4, 6B, 9V, 14, 18C, 19F and 23F. PCV10 (GSK) includes these serotypes, plus serotypes 1, 5 and 7F. Since the introduction of PCV in 2006, the uptake rate has remained high (>90%) [22], and after the PCV introduction, the incidence of IPD has declined in children (both<5 years and 5–17 year olds) and young adults (18–49 year olds) [23].

Despite the decline in IPD cases caused by vaccine serotypes (VTs), a subsequent increase in cases caused by non-vaccine serotypes (NVTs) erodes the long-term benefit of the vaccination programme [24]. After 12 years of paediatric PCV use in the Netherlands, a slight increase in non-PCV10 serotypes has been observed amongst IPD cases in children under 5 years of age, whilst the increase in adults aged 50 and above was more pronounced [23]. This was primarily due to the rapid expansion of a non-PCV10 serotype, serotype 8 and to a lesser extent by the non-PCV10 serotypes 9N, 12F and 6C in the adult population [23]. National surveillance of bacterial meningitis has demonstrated that the introduction of paediatric PCV vaccination was followed by a decrease in the proportion of cases caused by VTs in elderly adults. Nonetheless, the burden of adult bacterial meningitis caused by *S. pneumoniae* remains high [25].

Based on the observed antimicrobial resistance (AMR) profiles in the Netherlands, in 2024 empirical intravenous antimicrobial therapy of moderately severe community-acquired pneumonia (a major clinical syndrome related to IPD) is still confined to benzylpenicillin [26]. In 2023, 96% of pneumococcal meningitis cases in which antimicrobial susceptibility testing was performed could be treated with intravenous benzylpenicillin [16].

In this study, we performed whole-genome sequencing of 1013 pneumococcal blood isolates collected across all age groups in the Gelderland area, the Netherlands, between 2000 and 2020. We investigated genomic dynamics within this unbiased pneumococcal bacteraemia population alongside the introduction of paediatric immunization with PCVs and placed the local findings in a global context.

## Methods

### Study design

Of the 1013 pneumococcal isolates collected and sequenced, 1004 passed quality control. The unbiased population sample of * S. pneumoniae* blood isolates (*n*=979) was collected from consecutive patients with pneumococcal bacteraemia in three hospitals in the provinces of Gelderland and North Brabant: Canisius-Wilhelmina Ziekenhuis (Jan 2000 to Jun 2020; *n*=610), Radboudumc (Jan 2012 to Jun 2020; *n*=181) and Maasziekenhuis Pantein (Jan 2012 to Jun 2020; *n*=188) (Fig. S1, available in the online Supplementary Material). Retrospective study procedures were approved by the Medical Ethical committees of the participating hospitals, including a waiver for individual informed consent, file number 2020–6644 Radboudumc. Specifically in the study region, vaccine coverage was>90% throughout the study period, for children in the adherent region having completed the PCV 3+0 schedule at the age of 2 [27]. In 2020, the vaccination of adults with PCV+pneumococcal polysaccharide vaccine (PPV) was still confined to individuals with (functional) asplenia. An additional 25 pneumococcal isolates were included in this study due to their penicillin-resistant phenotype to relate their genetic content to the unbiased population sample. They were provided by the Netherlands Reference Laboratory for Bacterial Meningitis.

The 979 isolates in the unbiased population sample were grouped according to the vaccine period in which they were collected: pre-PCV (2000–2005), PCV7 (2006–2010), early-PCV10 (2011–2015) and late-PCV10 (2016–2020). For analysis by age, the isolates were grouped into children (<18 years old), adults (18–64 years old) and the elderly (≥65 years old) (Table 1).

**Table 1.** Number of pneumococcal isolates collected across the vaccine periods by age groups in Gelderland, the Netherlands, 2000–2020

Age group	Pre-PCV period	PCV7 period	Early-PCV10 period	Late-PCV10period
	2000–2005	2006–2010	2011–2015	2016 to June 2020
**<18 years old (*n*=48**)	10	9	14	15
**18–64yearsold (** ***n*** **=368)**	74	88	125	81
**≥65 years old (*n*=563)**	107	129	172	155
**Total (*n*=979)**	191	226	311	251

### Genome sequencing

The pneumococcal blood isolates were cultured overnight in 10 ml Todd-Hewitt broth+5% yeast extract at 37 °C and 5% CO_2_. Genomic DNA was extracted using the Qiagen genomic-tip/20 gravity columns. The Illumina HiSeq 2000 platform was used for the first 349 isolates and produced paired-end 100 bp reads. The rest of the collection was sequenced on the Illumina NovaSeq 6000 and produced paired-end 150 bp reads.

### Genome analyses

Genomes were assessed for quality control using the following parameters: sequencing depth of>20×; >60% reads mapped to reference genome *S. pneumoniae* ATCC 700669 (NCBI accession number FM211187); assembly length of 1.9–2.3 Mb, number of contigs<500 and number of heterozygous SNPs<220. Genomes that passed quality control were subject to *in silico* typing. For each genome, pneumococcal lineage or global pneumococcal sequencing cluster (GPSC) was assigned using PopPUNK v2.4.0 [28] using reference database (v6) [2]; *in silico* serotype and sequence type (ST) were inferred using SeroBA v1.0.2 [29] and mlst v2.22.0, respectively [30,31]. STs were then clustered into clonal complexes (CCs) using a six-loci cutoff as described previously [32].

AMR was predicted from genome data using the US Centres for Disease Control and Prevention pipeline [33,34], which generates predicted minimum inhibitory concentration (MIC) and categorizes the isolates into susceptible, intermediate or resistant according to CLSI guidelines (M100-ED28: 2018) [35]. Penicillin resistance was defined as MIC of≥0.12 µg ml^−1^, according to CLSI guidelines (Clinical and Laboratory Standards Institute; M100-ED28: 2018) meningitis breakpoints [35]. Antimicrobial susceptibility predictions were recorded in File S1.

A pseudo-genome alignment was created by mapping the reads of each genome in this study to the *S. pneumoniae* reference genome (ATCC_700669, Accession number: FM211187) using SMALT v.0.7.4. SNP sites were identified from the pseudo-genome alignment using snp-sites (v2.5.1) [36]. Phylogenetic analysis was performed on all genomes by constructing a maximum-likelihood tree using FastTree version v2.1.0 [37]. An interactive visualization of the phylogenetic tree and the associated metadata was created using Microreact https://microreact.org/project/gps2-netherlands. To investigate whether there was clonal expansion of penicillin-resistant isolates, we constructed GPSC-specific phylogenies of the major penicillin and/or amoxicillin-resistant GPSCs (GPSC6 and GPSC10). These were supplemented with genomes of the same GPSC from the global pneumococcal sequencing (GPS) project database (last accessed on 15 March 2023 [38]). To investigate the major multidrug-resistant (MDR) lineage GPSC1, GPSC1 genomes were gathered from the GPS project database. SKA2 (v1.0) [39] was used to generate an SNP alignment of the genome for each isolate. Recombination-free phylogenies for each of these GPSCs were constructed by Gubbins (v3.2.1) [40] using an input of alignment that was generated by mapping reads from each genome to their respective lineage-specific reference genomes [32]. Metadata, together with *in silico* typing results, were summarized in File S1.

### Statistical analysis

Changes in serotype and GPSC across the vaccine periods and age groups were analysed in the unbiased population sample of *S. pneumoniae* blood isolates (*n*=979).

Serotypes included in PCV7 (4, 6B, 9V, 14, 18C, 19F and 23F) and PCV10 (GSK; 1, 5 and 7F in addition to PCV7 serotypes) were considered VTs, and serotypes not included in these vaccines as NVTs. Differences in the proportion of cases attributable to NVTs between time periods or age categories were evaluated using the odds ratio of NVT over VT bacteraemia between two different time periods/age categories using the formula [(Number of NVT isolates in time A)/(Number of NVT isolates in time B)]/[(Number of VT isolates in time A)/(Number of VT isolates in time B)]. Odds ratios were calculated considering a 95% confidence interval and changes deemed significant if *P*-value<0.05. The statistical analysis was carried out in R version 4.1.2.

National surveillance of IPD in the Netherlands has clearly demonstrated serotype replacement during the study period [41]. As the current study aims to discern genomic dynamics within the declined VT population and the expanding NVT population, when looking at the changes for a given serotype, this was considered to be the number of cases caused by a specific serotype over the total number of either VT or NVT isolates, depending on which type the serotype of interest is. Therefore, changes in NVT serotypes are compared to the relative number of NVT cases, as opposed to cases in the total bacteraemia-causing population. Proportional changes in GPSC and serotype between vaccine periods and/or age groups were statistically tested in a two-sided fashion using a Fisher’s Exact test at a significance level of 0.05. The tests were performed using the R package pwr. Per analysis, multiple testing was adjusted for using the Benjamini–Hochberg false discovery rate at a control level of 5%.

To investigate the diversity of serotypes within each time period, Simpson’s diversity index was calculated using the R package vegan 2.6–8 [42]. Simpson’s diversity index reports infinite diversity (zero) to no diversity (one).

## Results and discussion

### Patient demographics

The 979 pneumococcal isolates were collected from consecutive positive blood cultures from patients aged between 0 and 102 years old in the Gelderland area, the Netherlands (Fig. S2). The paediatric fraction of the current study cohort (48/979=4.9%) matches national surveillance in 2023, where the 9 sentinel laboratories submitted a total of 28 paediatric blood isolates (0–19 years old) and 551 adult blood isolates (28/579=4.8%). This indicates that the number of paediatric IPD isolates in the study cohort is representative for the country [41]. Most of the isolates were from elderly individuals aged≥65 years old (*n*=563), followed by adults aged 18–64 years old (*n*=368) and children aged<18 years old (*n*=48) (Table 1). Note that missing cases may be present in the first 2 years of collection (2000–2001) because the identification of consecutive cases relied on paper documentation. In addition, the collection of isolates during the first two time periods was confined to one of the three hospitals involved. However, the serotype distribution does not appear to differ among the three hospitals in the last two time periods (Fig. S3), so it can be assumed that the serotype distribution in this one hospital was representative for the entire study area during the first two time periods.

### Pneumococcal serotypes and lineages

Among the 1004 pneumococcal isolates (979 from cases of bacteraemia, 25 from the Netherlands Reference Laboratory), 46 serotypes (Fig. S4) across 64 pneumococcal lineages or GPSCs were identified computationally (Fig. S5). Across the study period, the five most prevalent serotypes were serotype 8 (*n*=178; 17.7%), 7F (*n*=102; 10.2%), 3 (*n*=77; 7.7%), 19A (*n*=69; 6.9%) and 1 (*n*=66; 6.6%) (Fig. S4), collectively accounting for 49% (*n*=492/1004) of the isolates within the dataset. The dominant serotypes in our study sample correspond to those reported in national surveillance as performed by the Netherlands Reference Laboratory for Bacterial Meningitis [41]. For the Netherlands, Vissers *et al.* [43] and Vestjens *et al.* [23] also described the expansion of serotype 8 in a paediatric carriage as well as adult IPD post-PCV introduction. A high prevalence of serotype 8 in IPD samples has been observed in other countries post-PCV13, such as Denmark [44], Portugal [45], Argentina [46] and South Africa [47].

Among the 64 GPSCs within the dataset, the dominant lineages were GPSC3 (CC53 expressing serotype 8, Netherlands^8^-33 PMEN clone), GPSC15 (CC191 expressing serotype 7F, Netherlands^7F^-39), GPSC12 (CC180 expressing serotype 3, Netherlands^3^-31), GPSC31 (CC306 expressing serotype 1, Sweden^1^-28) and GPSC19 (CC433 expressing serotype 22F), constituting 49% (493/1004) of the entire collection (Fig. S5). We also noted that serotype 19A, one of the most prevalent serotypes within the dataset, was expressed by multiple GPSCs. In total, 44.9% of serotype 19A isolates were members of GPSC4 (*n*=31/69), followed by 10.1% of isolates each in GPSC10 and GPSC146, with the remaining isolates split between nine other GPSCs (*n*=24/69; 34.8%).

**Fig. 1.**
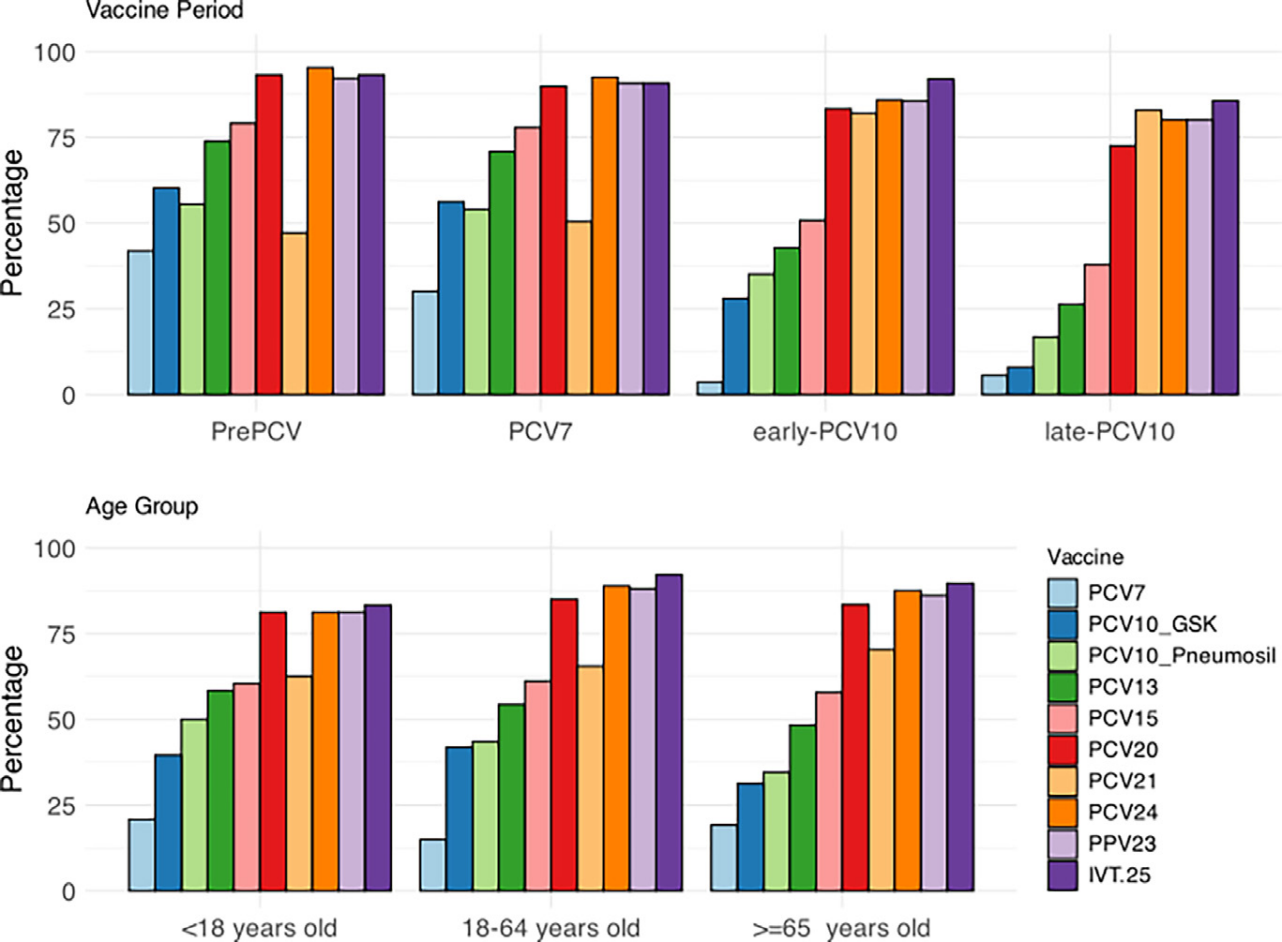
Coverage of circulating serotypes in Gelderland, Netherlands, by various vaccine formulations. (Top) Serotype coverage over the different vaccine periods: pre-PCV (2000–2005), PCV7 (2006–2010), early-PCV10 (2011–2015) and late-PCV10 (2016–2020). (Bottom) Serotype coverage by age groups over a 20-year time period. At the time of writing, PCV15 and PCV20 were approved to be used in children and adults; PCV21, PCV24 and IVT-25 were not approved to be used in adults and children and were still under development.

Fig. 1 demonstrates serotype coverage by pneumococcal vaccines for the 20-year population sample by vaccine periods and age groups. Most cases concern adults, and in the late-PCV10 era, we observe that coverage of overall pneumococcal bacteraemia cases by PCV7 and PCV10 (GSK) dropped below 10%. This most recent time period (2016–2020) also demonstrates a clear increase in serotype coverage from PCV13 (<30%) to PCV20 (>70%) and other pneumococcal vaccines with higher valencies. There were no major differences in serotype coverage over the 20-year study period between the different age groups, aside from lower coverage by PCV10 (GSK) and PCV13 in the elderly population [PCV10 (GSK): 57.9% coverage in the elderly pre-PCV; 5.8% coverage in the elderly in the late-PCV10 period. PCV13: 72% coverage in the elderly pre-PCV; 25.2% coverage in the elderly in the late-PCV10 period], alongside increasing coverage by PCV21 in that group (49.5% coverage in the elderly pre-PCV; 85.2% coverage in the elderly in late-PCV10).

### Changes in VT and NVT levels

**Fig. 2.**
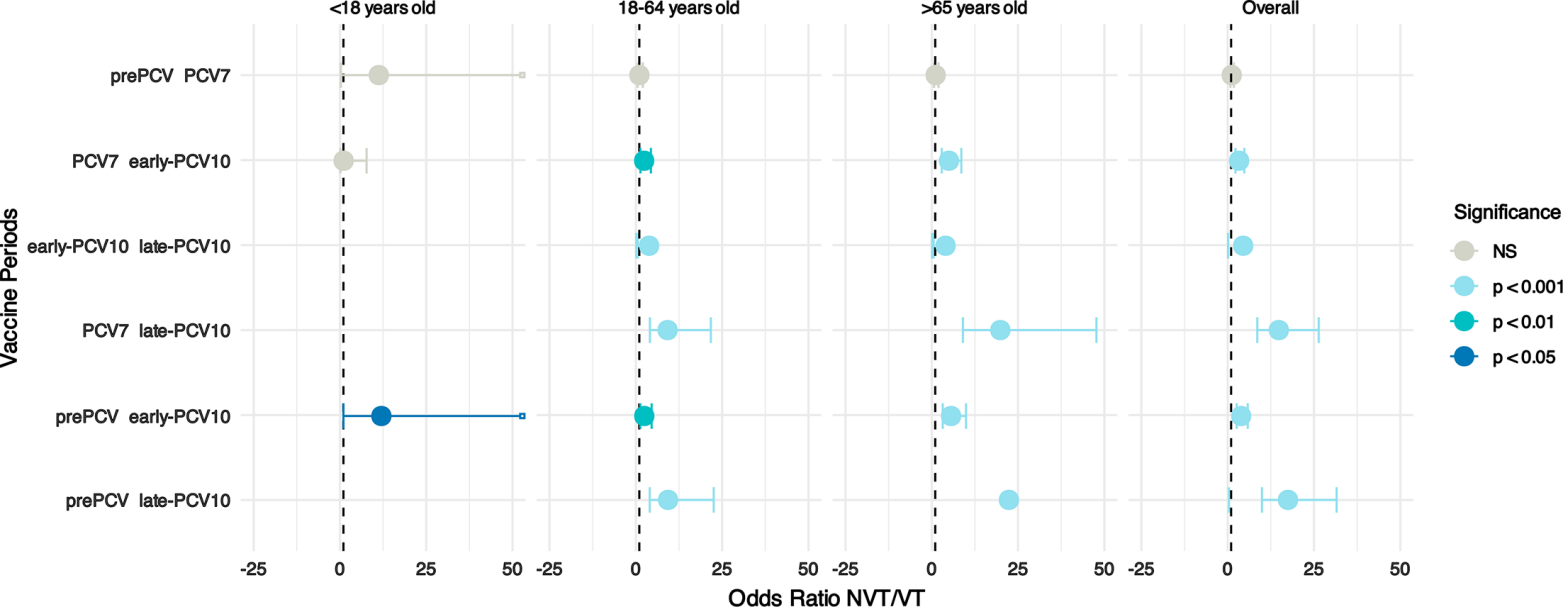
Changes in bacteraemia cases caused by non-PCV10 serotypes relative to PCV10 serotypes in the Gelderland area, the Netherlands, coloured by statistical significance. Some confidence intervals (marked with hollow squares) extend beyond the range of the *x*-axis. Odds ratios are not shown where the confidence intervals were infinite due to low numbers of samples. An odds ratio of>1 shows an increase in non-PCV10 serotype samples compared to PCV10 serotype samples. The results are shown for different vaccine periods, defined as pre-PCV (2000–2005), PCV7 (2006–2010), early-PCV10 (2011–2015) and late-PCV10 (2016–2020).

To investigate the increase in the proportion of NVTs causing bacteraemia compared to VTs, we calculated the odds ratio for NVT over VT pneumococcal isolates across the indicated time periods. Compared to the pre-PCV period, the odds ratio for NVT bacteraemia was 17.1 (CI 9.7–30.8; *P*<0.001) in the late-PCV10 period, showing an overall increase in the proportion of bacteraemia cases being caused by NVT pneumococci (Fig. 2).

To investigate how this varied by patient age, the data were stratified into age groups. The limited number of paediatric cases (<18 years old) only partially allowed for statistical analysis. A high uptake rate of PCV7 and PCV10 in children [48] was followed by a decrease in the proportion of VTs causing IPD in children as well as in the non-vaccinated adult populations (Fig. 2).

### Changes in serotype across vaccine periods by age

**Fig. 3.**
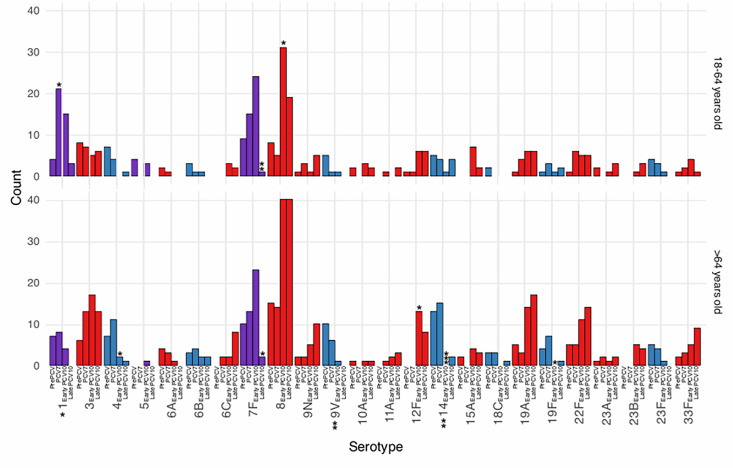
Number of pneumococcal serotypes associated with bacteremia across vaccine periods, stratified by age groups in the Gelderland area, the Netherlands, 2000–2020. Red denotes non-PCV10 (GSK) serotypes; blue denotes serotypes covered by PCV7; purple denotes serotypes covered by PCV10, but not PCV7. Significance levels are compared to the same serotype in the previous vaccine period. Significant markers at the *x*-axis represent significant changes between the pre-PCV and late-PCV10 time periods (*, *P*<0.05; **, *P*<0.001; ***, *P*<0.0001). Serotypes with less than ten isolates were not included in the figure. The>18 years group is not included in the figure because the number of cases in this unbiased population sample was insufficient to reach statistical power for individual serotypes.

In children, we observed an increasing proportion of NVT isolates over time, although the number of cases in this unbiased population sample was insufficient to reach statistical power for individual serotypes (Fig. 3). In the late-PCV10 period, solely NVT pneumococcal bacteraemia was observed in paediatric cases (*n*=15).

Overall, considering each serotype in the overall population in the late-PCV10 period, the VTs 7F (*P*<0.0001), 9V (*P*=0.04), 4 (*P*=0.009), 14 (*P*=0.01) and 23F (*P*=0.04) had significantly decreased, but NVTs 8 (*P*=0.0002), 12F (*P*=0.008) and 23B (*P*=0.04) had significantly increased when compared to the PCV7 period.

In the adult age group (18–64 years old), we noticed a significant increase in the proportion of NVT cases caused by serotype 8 after the introduction of PCVs into the paediatric immunization schedule (PCV7=13.9% of NVTs are serotype 8, early-PCV10=39.7% of NVTs are serotype 8, *P*<0.05). In the elderly, serotype 8 had already been the major serotype pre-PCV and remained dominant, aside from a temporary transcendence by serotype 14 in the PCV7 period. Concerningly, serotype 8 was previously noted to be an invasive serotype in a meta-analysis [49].

After the introduction of PCV7, there was a significant increase in serotype 1 in the adult age group in this study. Although previous studies [50,51] have noted an increase in serotype 7F in both the adult and elderly age groups in the Netherlands, we did not see statistically significant increases in either age groups in this study (Fig. 3). Serotypes 1 and 7F are not included in the PCV7 vaccination but are in the PCV10 vaccination. After the introduction of PCV10 in children, we first observed a decrease in serotype 1 bacteraemia, later followed by a significant drop in 7F bacteraemia in both adults and elderly only in the late-PCV10 period. This is in line with a previous study that showed that the pneumococcal population stabilized ~7 years after vaccine introduction in Massachusetts [52].

In general, in the elderly age group (≥65 years old), changes in serotype distribution were not as pronounced in the first 5 years after vaccine introduction, and there were no significant differences between the pre-PCV period and the PCV7 period (Fig. 3). Later, however, between the PCV7 and early-PCV10 periods, we observed a significant decrease in the PCV7 VTs serotype 14 (*P*<0.0001), serotype 4 (*P*<0.05) and serotype 19F (*P*<0.05). At the same time, there was a significant increase in NVT serotype 12F (*P*<0.05), which was not seen in any other age group. Concerningly, 12F has been associated with IPD outbreaks in adults in prisons in the USA [53], children in Japan [54] and adults in Canada [55]. Overall, it appears that serotypes 8, in the adult age group, and 12F, in the elderly, were increasing.

Finally, we investigated whether or not there had been a change in the diversity of serotypes causing bacteraemia over the study period. We found that there were no significant differences in Simpson’s diversity index between the time periods, with all time periods having high score (pre-PCV=0.93, PCV7=0.93, early-PCV10=0.89, late-PCV10=0.90), showing low diversity of serotypes within each time period.

### Changes in lineage across vaccine periods by age

We looked at changes in the distribution of *S. pneumoniae* lineages over the course of the study by age. As there were few cases in the≤18 year old group, there were no significant changes in lineages detected across the vaccine periods (Fig. S6). Differences could be seen in the distribution of GPSC lineages across the vaccine periods for both the adult (Fig. S7A) and elderly (Fig. S7B) populations. However, significant changes were only seen in the adult group (Table 2). Between the PCV7 period and the early-PCV10 period, there was a significant increase in the proportion of GPSC3 (*P*=0.04) isolates. There was also a significant increase in GPSC31 (containing only serotype 1, *P*=0.04) between the pre-PCV and PCV7 periods, followed by a significant decrease in the late-PCV10 period likely due to the inclusion of serotype 1 in the PCV10 vaccination. Finally, there was also a significant decrease in the proportion of GPSC27 (containing only serotype 4, *P*=0.04) isolates between the pre-PCV period and the early-PCV10 period.

**Table 2.** Distribution in number and percentage of GPSCs found in the pneumococcal population from the Gelderland area in the<18 years old, 18–64 years old and≥65 years old age groups across the pre-PCV, PCV7, early-PCV10 and late-PCV10 vaccine periods. The total number of isolates within each age group is indicated and stratified by vaccine period

	<18 years old (*n*=48)				18–64 years old (*n*=368)				≥65 years old (*n*=563)			
	**Pre-PCV (*n*=10)**	**PCV7 (*n*=9)**	**Early-PCV10 (*n*=14)**	**Late-PCV10 (*n*=15)**	**Pre-PCV(*n*=74)**	**PCV7 (*n*=88)**	**Early-PCV10 (*n*=125)**	**Late-PCV10 (*n*=85)**	**Pre-PCV(*n*=107)**	**PCV7 (*n*=129)**	**Early-PCV10 (*n*=172)**	**Late-PCV10 (*n*=155)**
**GPSC**												
3	1 (10%)	1 (11%)	2 (14%)	4 (27%)	9 (12%)	8 (9%)	34 (27%)^*a*^	22 (27%)	16 (15%)	18 (14%)	46 (27%)	49 (32%)
15	–	2 (22%)	3 (21%)	–	8 (11%)	13 (15%)	24 (19%)	–	10 (9%)	13 (10%)	23 (13%)	2 (1%)
12	–	–	–	2 (13%)	6 (8%)	4 (5%)	5 (4%)	5 (6%)	5 (5%)	12 (9%)	15 (9%)	13 (8%)
31	1 (10%)	–	3 (21%)	–	4 (5%)	21 (24%)^*b*^	15 (12%)	3 (4%)^*c*^	7 (7%)	8 (6%)	4 (2%)	–
19	–	–	–	–	1 (1%)	6 (7%)	5 (4%)	5 (6%)	4 (4%)	5 (4%)	11 (6%)	14 (9%)
7	–	–	–	2 (13%)	6 (8%)	3 (3%)	3 (2%)	5 (6%)	5 (5%)	6 (5%)	6 (3%)	6 (4%)
4	–	–	3 (21%)	1 (7%)	–	4 (5%)	3 (2%)	4 (5%)	2 (2%)	6 (5%)	4 (2%)	9 (6%)
27	1 (10%)	–	–	–	7 (9%)	4 (5%)	–^*d*^	–	7 (7%)	11 (9%)	2 (1%)	1 (1%)
6	–	–	1 (7%)	–	5 (7%)	2 (2%)	1 (1%)	1 (1%)	10 (9%)	7 (5%)	5 (3%)	1 (1%)
16	–	–	–	–	1 (1%)	4 (5%)	2 (2%)	5 (6%)	3 (3%)	2 (2%)	4 (2%)	10 (7%)
18	3 (30%)	2 (22%)	–	1 (7%)	3 (4%)	2 (2%)	–	1 (1%)	6 (6%)	6 (5%)	–	–
39	–	–	–	–	1 (1%)	2 (2%)	1 (1%)	3 (4%)	9 (8%)	9 (7%)	–	2 (1%)
32	–	–	1 (7%)	–	1 (1%)	3 (3%)	3 (2%)	1 (1%)	1 (1%)	–	6 (3%)	2 (1%)
24	1 (10%)	–	–	–	2 (3%)	1 (1%)	1 (1%)		3 (3%)	5 (4%)	1 (1%)	3 (2%)
26	–	–	–	1 (7%)	–	–	–	3 (4%)	–	–	8 (5%)	4 (3%)
29	–	–	–	–	1 (1%)	–	2 (2%)		1 (1%)	–	2 (1%)	6 (4%)
44	1 (10%)	–	–	–	–	1 (1%)	1 (1%)	3 (4%)	1 (1%)	3 (2%)	–	2 (1%)
10	–	–	–	–	1 (1%)	2 (2%)	3 (2%)		2 (2%)	1 (1%)	1 (1%)	1 (1%)
11	–	1 (11%)	–	1 (7%)	–	–	3 (2%)	–	–	2 (2%)	2 (1%)	2 (1%)
35	–	–	–	1 (7%)	2 (3%)	–	2 (2%)	2 (3%)	1 (1%)	–	3 (2%)	–
50	1 (10%)	–	–	–	2 (3%)	–	–	–	3 (3%)	2 (2%)	1 (1%)	1 (1%)
36	–	–	–	–	–	2 (2%)	–	1 (1%)	–	1 (1%)	3 (2%)	3 (2%)
Others	1 (10%)	3 (33%)	1 (7%)	2 (13%)	14 (19%)	6 (7%)	18 (14%)	18 (22%)	13 (12%)	13 (10%)	25 (15%)	

*a* Significant increase of the GPSC in the early-PCV10 when compared to the PCV7 period *P*<0.05. Significant increase of the GPSC in the PCV7 period when compared to the Ppre-PCV *P*<0.05. Significant decrease of the GPSC in the late-PCV10 when compared to the PCV7 *P*<0.05. Significant decrease of the GPSC when compared to the Ppre-PCV period.

We identified serotype 8, which increased significantly in adults between the PCV7 and early-PCV10 periods, and serotype 12F, which increased significantly in the elderly between the PCV7 and early-PCV10 periods as emerging serotypes, as they are the only NVT serotypes that are seen to significantly increase over the time period of the study. We decided to investigate the lineages driving these emerging serotypes. Overall, 94.9% of serotype 8 isolates in the dataset belonged to GPSC3 (*n*=169/178), with 3.9% in GPSC98 and 0.6% each in GPSC32 and GPSC694. The majority of serotype 8 samples belong to ST53 (*n*=165/178; 93%), consistent with findings from studies in Denmark [44], Portugal [56], the United Kingdom [11], South Africa [4,57] and Spain [58], which also reported a marked increase in ST53 serotype 8 in adults post-vaccine introduction (Fig. S8).

We investigated the changes in the lineages expressing serotype 8 over the course of the study to see if there were any significant changes in the dominant lineage with the introduction of PCVs. In the pre-PCV period, the serotype 8 isolates in the dataset belonged to GPSC3 (*n*=21/23; 91.3%), GPSC32 (*n*=1/23; 4.3%) and GPSC98 (*n*=1/23; 4.3%). Over time, GPSC3 samples significantly increased (PCV7 vs. early-PCV10, *P*=0.002; PCV7 vs. late-PCV10, *P*=0.0001). In the late-PCV10 period, the serotype 8 lineages were GPSC3 (*n*=58/62; 93.5%) and, to a lesser extent, GPSC98 (*n*=4/62; 6.5%). In the dataset, GPSC3 contained multiple different serotypes alongside dominant serotype 8 (*n*=169; 80.5%). These included 33F (*n*=28; 13.3%), 11A (*n*=10; 4.8%), 11E (*n*=2; 0.95%) and 22F (*n*=1; 0.48%). All of these are NVTs. However, GPSC98 contained only serotype 8.

We performed a similar analysis for lineages expressing serotype 12F: GPSC32 (children, *n*=1; adult, *n*=8; elderly, *n*=9), GPSC26 (children, *n*=1; adult, *n*=3; elderly, *n*=12) and GPSC55 (children, *n*=0; adult, *n*=6; elderly, *n*=2). In the pre-PCV and PCV7 periods, all serotype 12F pneumococci belonged to GPSC32. In the late-PCV10 period, 53% (8/15) of the serotype 12F bacteraemia cases were from lineage GPSC26, 33% (5/15) from GPSC55 and 14% (2/15) from GPSC32. Globally, the majority of isolates belonging to GPSC26 (91%, *n*=177/195) [59] and GPSC55 (100%, *n*=90/90) [60] expressed serotype 12F, while GPSC32 expressed 12F (53%, *n*=66/124) alongside serotypes 7F (43%, *n*=53/124), 8 (2.4%, *n*=3/124) and 9N (1.6%, *n*=2/124) [61]. Microreacts for GPSC26, GPSC55 and GPSC32 can be found at https://microreact.org/project/gpsGPSC26, https://microreact.org/project/gpsGPSC32 and https://microreact.org/project/gpsGPSC55.

### AMR prevalence across vaccine periods by age group

Of the 979 isolates collected in the unbiased population sample, 127 displayed resistance to at least one class of antibiotic based on the antimicrobial susceptibilities predicted from the genome data (Fig. S9). We also detected 60 isolates across 16 GPSCs that were MDR, predicted non-susceptible to at least three classes of antibiotics (Table S2). However, the overall prevalence of antibiotic resistance was low, likely because as of 2014, the Netherlands had one of the lowest antibiotic consumption rates in Europe [62]. We further investigated changes in AMR before and after PCV introduction by stratifying the data by vaccine period and age (Table S1). There were no significant changes in AMR prevalence after vaccine introduction (*P*>0.05 for all comparisons).

The overall prevalence of antibiotic resistance by age group was summarized in Table S1. Statistically significant differences were seen between the age groups for penicillin (*P*=0.041), with greater proportions of resistant isolates in adults, and erythromycin (*P*=0.039), which had greater proportions of resistant isolates in children. In children, the highest proportion of resistance was seen to erythromycin (*n*=7/48; 14.5%), whilst in the adult and elderly population, resistance to erythromycin was much lower (*n*=19/368, 5.2% in adults; *n*=29/563, 5.2% in the elderly). Erythromycin-resistant samples in children were only isolated in the pre-PCV and PCV7 time periods, belonged to VTs and NVTs, and were identified as GPSC18 (*n*=4), GPSC24 (*n*=1) and GPSC99 (*n*=2). The children affected by these macrolide-resistant isolates had no medical conditions that were associated with the use of macrolides, and they did not take macrolides at the time of hospital admission. In primary healthcare settings in the Netherlands, current guidelines do not support the use of macrolides as a first-line choice for paediatric infections. In 2015, the defined daily doses (DDD) for macrolide use in the Netherlands was 434 per 1000 population; it was lower compared to other countries in Western Europe in 2015 (Germany, DDD=916; Denmark, DDD=664; United Kingdom, DDD=1218; France, DDD=1311; Belgium, DDD=1379) [63]. Therefore, the lack of detection of macrolide resistance in the current paediatric bacteraemia sample was more likely attributable to the confined use of antibiotics or the low number of paediatric samples than to the effects of pneumococcal vaccination. In adults and the elderly, the highest proportion of resistant isolates was observed for cotrimoxazole (*n*=37/368, 10.1% in adults; *n*=55/563, 9.8% in the elderly).

The first-line treatments for moderately severe pneumonia in the Netherlands are benzylpenicillin and amoxicillin. Penicillin and amoxicillin were among the antibiotics with the lowest proportion of resistant isolates in the dataset based on the prediction from the genome; only one isolate in the unbiased population sample was resistant to penicillin at the non-meningitis breakpoint (0.1%). An additional 31 isolates (3.3%) had reduced susceptibility to penicillin (MIC range 0.12–0.5 mg l^−1^) but were only resistant at the meningitis breakpoint. The majority of these patients had either a severely immunocompromising condition, had used antibiotics within the 48 h prior to admission, lived abroad, or had a chronic respiratory condition. Three of those bacteraemic patients were diagnosed with meningitis and had been empirically treated with intravenous broader spectrum beta-lactam antibiotics according to national antibiotic guidelines. The adult population had a higher resistance to penicillin at the meningitis breakpoint (*n*=19/368; 5.2%) compared to children (*n*=1/48; 2.1%) and the elderly (*n*=12/563; 2.1%). In 2015, the DDD per 1000 population for broad-spectrum penicillins was 1868, which is lower compared to other countries in Western Europe (Germany, DDD=2092; United Kingdom, DDD=3320; France, DDD=8357; Belgium, DDD=8405) [49], which could explain this lack of resistance. Of note, serotype 8 isolates, which we identified as an emerging serotype, were not resistant to penicillin and amoxicillin (Fig. S9). Despite a reported sample of pneumococci isolated from diagnostics in the primary care setting that possibly indicated an increase in penicillin resistance in the Netherlands [64], our dataset does not show an increase in penicillin MIC based on genotype (Fisher’s Exact test comparing year of collection and predicted penicillin MIC, *P*=0.51) (Fig. S10). However, long-term monitoring is needed to ensure that the prevalence of AMR is not increasing and risk making current treatment plans ineffective.

The currently low prevalence of AMR does not exclude the emergence of this concern in the future. Therefore, we investigated which lineages contributed to AMR; specifically, we investigated the isolates within the bacteraemia dataset with predicted reduced susceptibility to penicillin or amoxicillin (i.e. only resistant at the meningitis breakpoint; *n*=32), as these need clinical consideration given the use of these antibiotics as first-line treatment in pneumonia. These isolates were collected between 2001 and 2020, throughout the whole time period of the study. They belonged primarily to lineages GPSC10 (*n*=11; 34.4%), GPSC47 (*n*=6; 18.8%), GPSC5 (*n*=3; 9.4%) and GPSC9 (*n*=3; 9.4%). There was a single penicillin-resistant isolate each in GPSC1, GPSC7, GPSC13, GPSC16, GPSC17, GPSC18, GPSC29, GPSC44 and GPSC55 (*n*=9; 28.1%). As an emerging pneumococcal lineage, GPSC3 isolates were susceptible to penicillin and amoxicillin. Interestingly, in the penicillin-resistant pneumococcal blood culture isolates from the Netherlands Reference Laboratory for Bacterial Meningitis (all MIC>2 mg l^−1^), they belonged to GPSC6 (*n*=8; 32%), GPSC1 (*n*=7; 28%), GPSC16 (*n*=3; 12%), GPSC9 (*n*=2; 8%) and a single isolate each in GPSC3, GPSC5, GPSC10, GPSC26 and GPSC81 (*n*=5; 20%).

From this, we further investigated the largest lineages with reduced penicillin susceptibility in the unbiased population sample (GPSC10) and in the penicillin-resistant isolates from the Netherlands Reference Laboratory (GPSC6). We constructed global phylogenies for GPSC6 (https://microreact.org/project/gps6-nl-global-context) and GPSC10 (Fig. S11) (https://microreact.org/project/gpsc10-nl-global-context) using additional data from the GPS project database [38]. In these lineages, isolates from the Netherlands did not form a single monophyletic clade but instead demonstrated closer relatedness to isolates from other countries. This makes it unlikely that pneumococcal blood isolates in the Netherlands with reduced susceptibility to penicillins have clonally expanded, but rather originated from multiple introductions of these lineages into the Gelderland area, Netherlands. Globally, GPSC1 represented a lineage of concern, as it only exhibited multidrug resistance, including penicillin resistance. Although this lineage was identified only once in the unbiased population sample, it accounted for 28% (*n*=7/25) of the isolates collected for their penicillin resistance phenotype by the Netherlands Reference Laboratory for Bacterial Meningitis. GPSC1 was frequently associated with serotype 19A, which remains one of the major serotypes causing IPD in the Netherlands. To investigate these isolates, we constructed a phylogeny for GPSC1 using data from the GPS1 dataset (https://microreact.org/project/gpsc1-nl-global-context) (Fig. S12). Similarly to GPSCs 6 and 10, we also saw that the isolates from the Netherlands did not form a single monophyletic clade, again showing that these concerned separate introductions of MDR GPSC1 to the Netherlands.

There are limitations of the current study: (1) the study was concluded in 2020, prior to the introduction of PPV23 vaccination in the elderly population; (2) while the unbiased population sample was representative for national surveillance of blood culture isolates, it did not include IPD captured from other sterile body sites, and it did not yet allow for in-depth genomics analyses in the paediatric IPD population. However, this stable local study cohort that includes bacterial genomics and clinical metadata supports further monitoring and interpretation of IPD dynamics. Moreover, this study demonstrates that context-specific concerns can be clarified by relating local dynamics to specified international datasets.

## Conclusion

In summary, this study describes the serotype and genotype distribution of pneumococci causing bacteraemia in the era of infant PCV use in the Gelderland area, the Netherlands. Serotype 8 has been identified as the primary cause of bacteraemia in the Gelderland area across the different age groups, with serotype 12F also posing a concern for the elderly in particular. The inclusion of serotypes 8 and 12F in PCVs of 20-valency and higher suggests their potential benefit, aligning with previous European studies that highlight PCV20’s effectiveness in addressing serotype 8 in adult IPD cases [65]. The dataset revealed a low prevalence of AMR to first-line antibiotics like penicillin and amoxicillin but underscored the need for continued monitoring, especially for GPSC6, GPSC10 and GPSC1 lineages, which exhibited higher proportions of resistant isolates. Whilst the introduction of PCV7 and PCV10 in children significantly reduced VT IPD, shifting the burden towards NVTs, this study emphasizes the need for targeted adult and elderly vaccination programmes, as the majority of cases occurred in these age groups. These findings reinforce the importance of genomic surveillance for vaccination strategies.

## supplementary material

10.1099/mgen.0.001377 Uncited Fig. S1.

## Abbreviations

AMO: amoxicillin
CFX: cefuroxime
CHL: chloramphenicol
CLI: clindamycin
COT: cotrimoxazole
DDD: defined daily doses
DOX: doxycycline
ERY: erythromycin
GPSCs: global pneumococcal sequencing clusters
IPD: invasive pneumococcal disease
MER: meropenem
NVT: non-vaccine serotype
PCV: pneumococcal conjugate vaccine
PCV7: 7-valent pneumococcal conjugate vaccine
PCV10: 10-valent pneumococcal conjugate vaccine
PEN: penicillin
TAX: cefotaxime
TET: tetracycline
VTs: vaccine serotypes
WGS: whole-genome sequencing
