# Supplementary material for: Investigating two decades of Streptococcus pneumoniae bacteraemia in the Gelderland area, the Netherlands, using whole-genome sequencing
Source: Microb Genom. 2025 Mar 18;11(3):001377. doi: 10.1099/mgen.0.001377 (PMC11936379; doi:10.1099/mgen.0.001377)
Supplement: Uncited Fig. S1. [file mgen-11-01377-s001.pdf]

Supplementary Figures

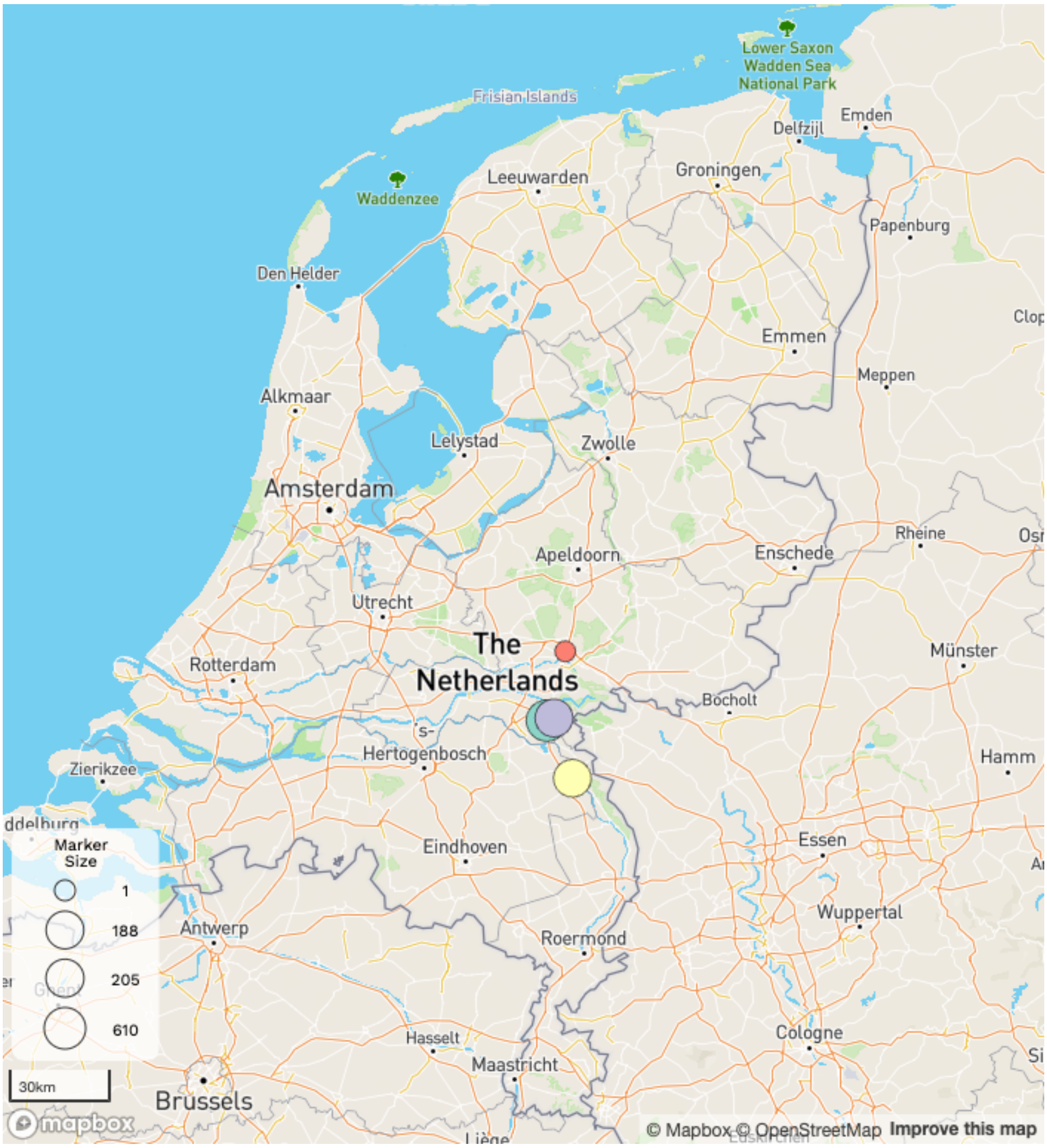

Figure S1 - Geographical map showing the location of the facilities from where the isolates were recovered. Canisius-Wilhelmina Ziekenhuis (green colour), Radboudumc (grey colour), Maasziekenhuis Pantein (yellow colour) and Rijnstate (orange colour). The markers in the map are scaled to reflect the number of isolates recovered in that location.

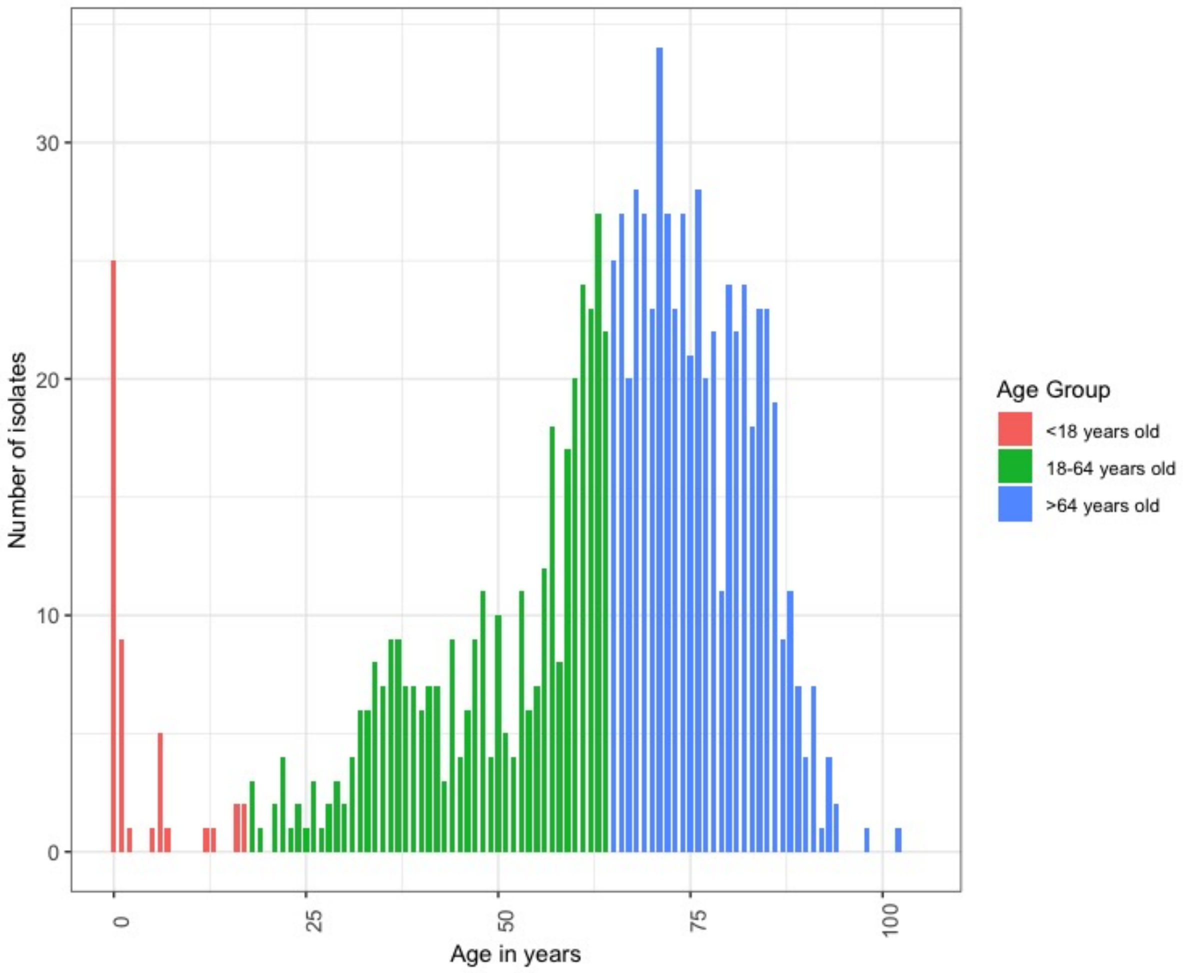

Figure S2 - Age distribution of 979 patients with bacteremia caused by Streptococcus pneumoniae in this study. The pneumococcal isolates were recovered in the Gelderland area, The Netherlands between 2000 and 2020. The bars indicate the number of pneumococcal isolates recovered among the different age groups <18 years old in red, 18 to 64 years in green and ≥65 years old in blue. The figure does not include the 24 penicillin resistance isolates from the National Reference Centre because host age and year of collection were unavailable for these isolates.

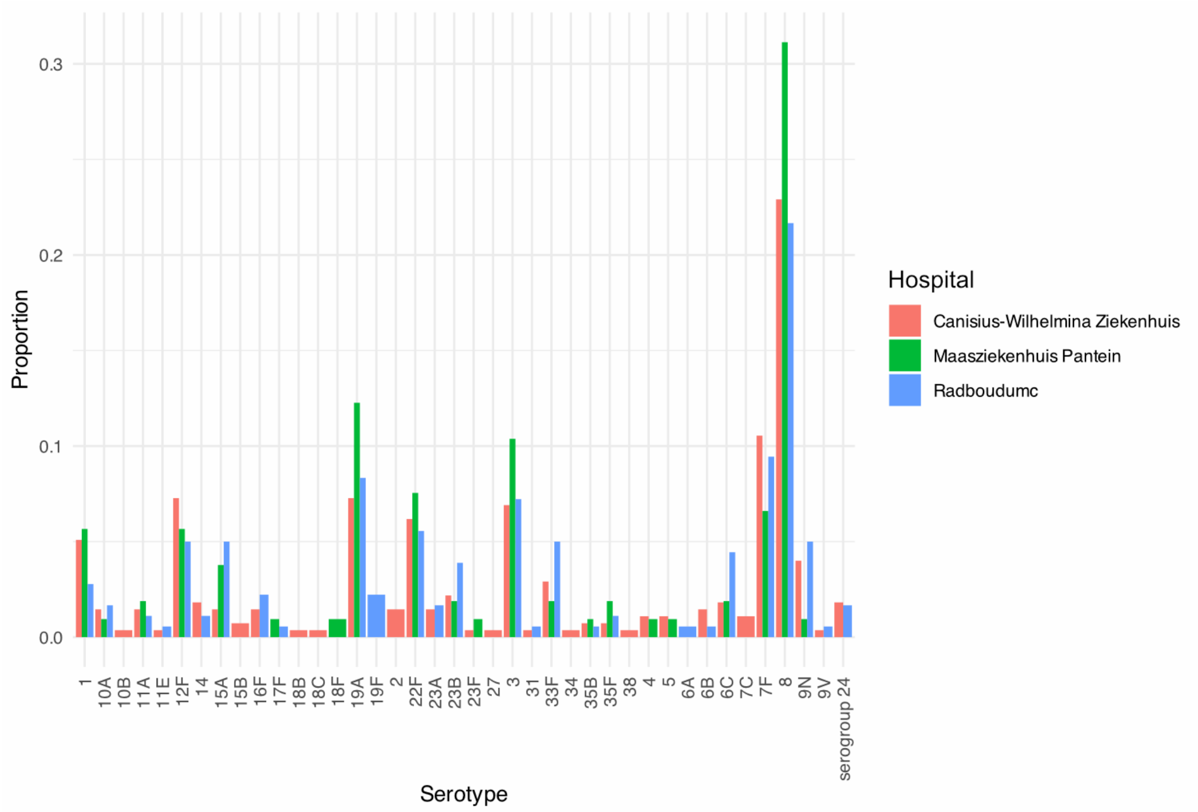

Figure S3 - The distribution of serotypes detected from bacteraemia cases in the three hospitals in the Gelderland area, The Netherlands between 2000 and 2020.

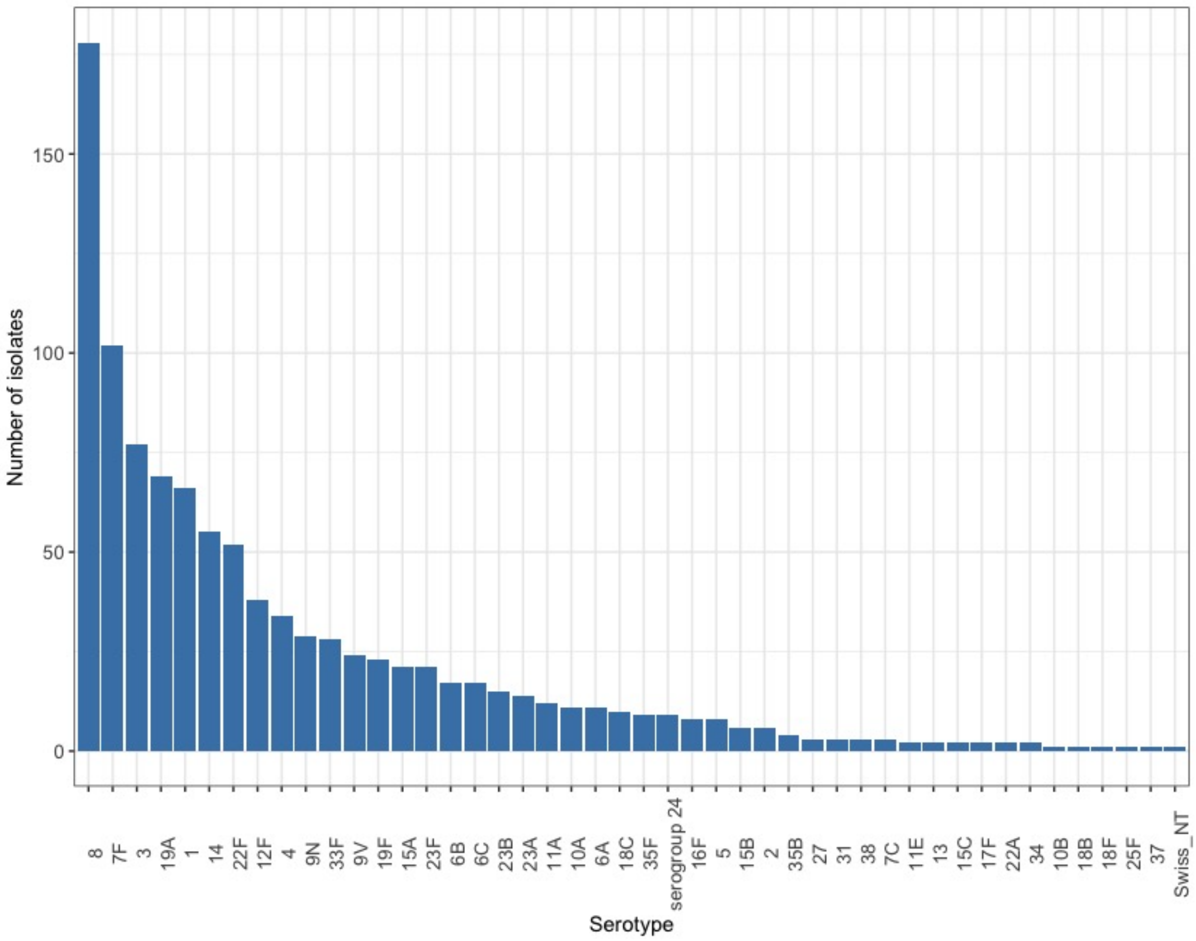

Figure S4 - Overall serotype distribution of 1004 pneumococcal isolates from blood culture positive clinical sample the Gelderland area, The Netherlands (n=979) and the penicillin-resistant pneumococcal blood culture isolates from the Netherlands Reference Laboratory for Bacterial Meningitis (n=25).

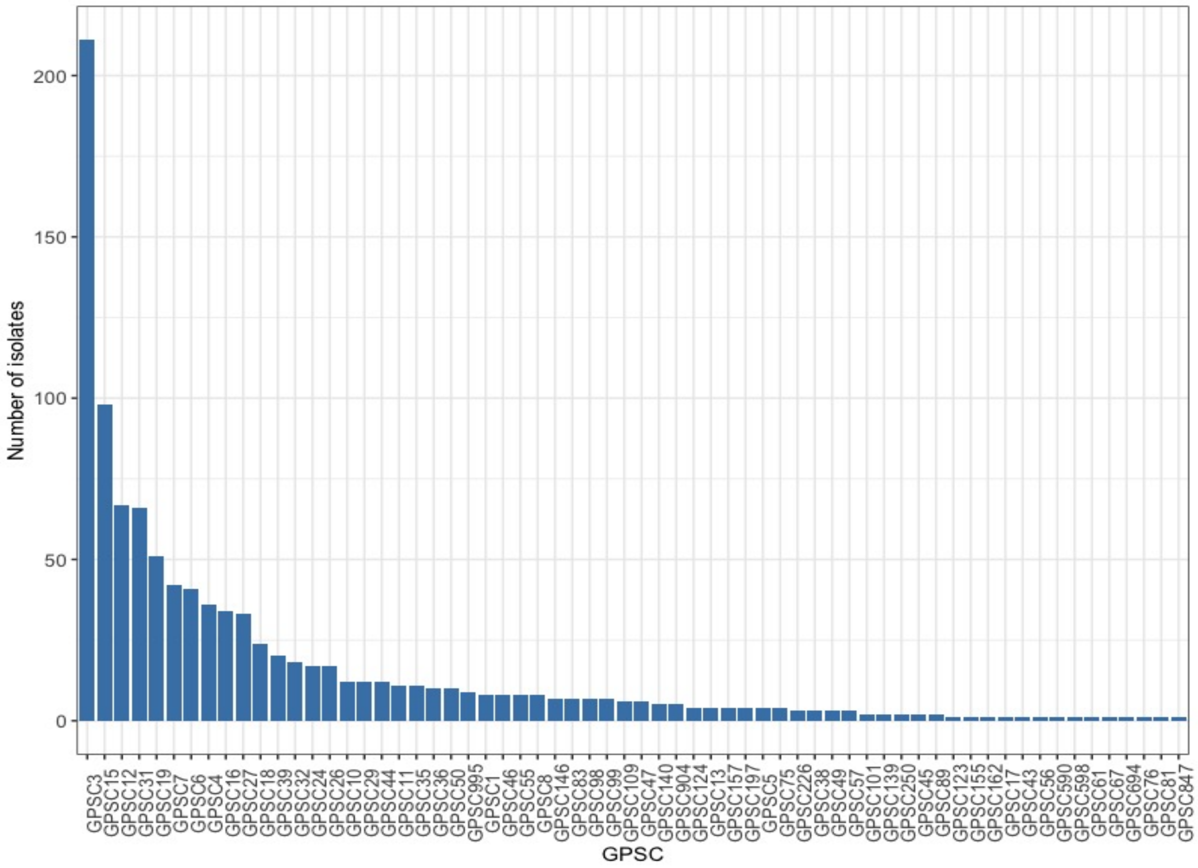

Figure S5 - Overall Global Pneumococcal Sequence Cluster (GPSC) distribution of 1004 pneumococcal isolates from blood culture positive clinical sample the Gelderland area, The Netherland (n=979) and the penicillin-resistant pneumococcal blood culture isolates from the Netherlands Reference Laboratory for Bacterial Meningitis (n=25).

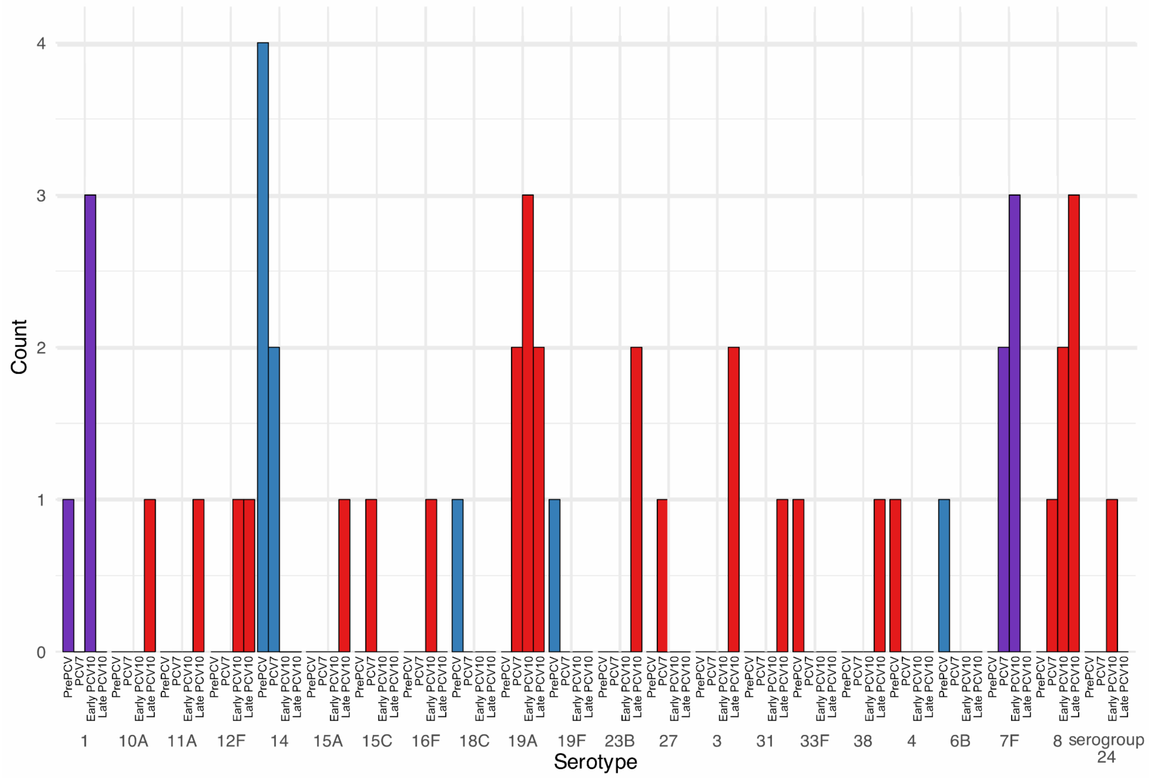

Figure S6 - Distribution of Serotypes across vaccine periods for samples collected from children (<18 years old). Blue serotypes are VTs, red are NVTs, and purple are serotypes in PCV10 but not in PCV7.

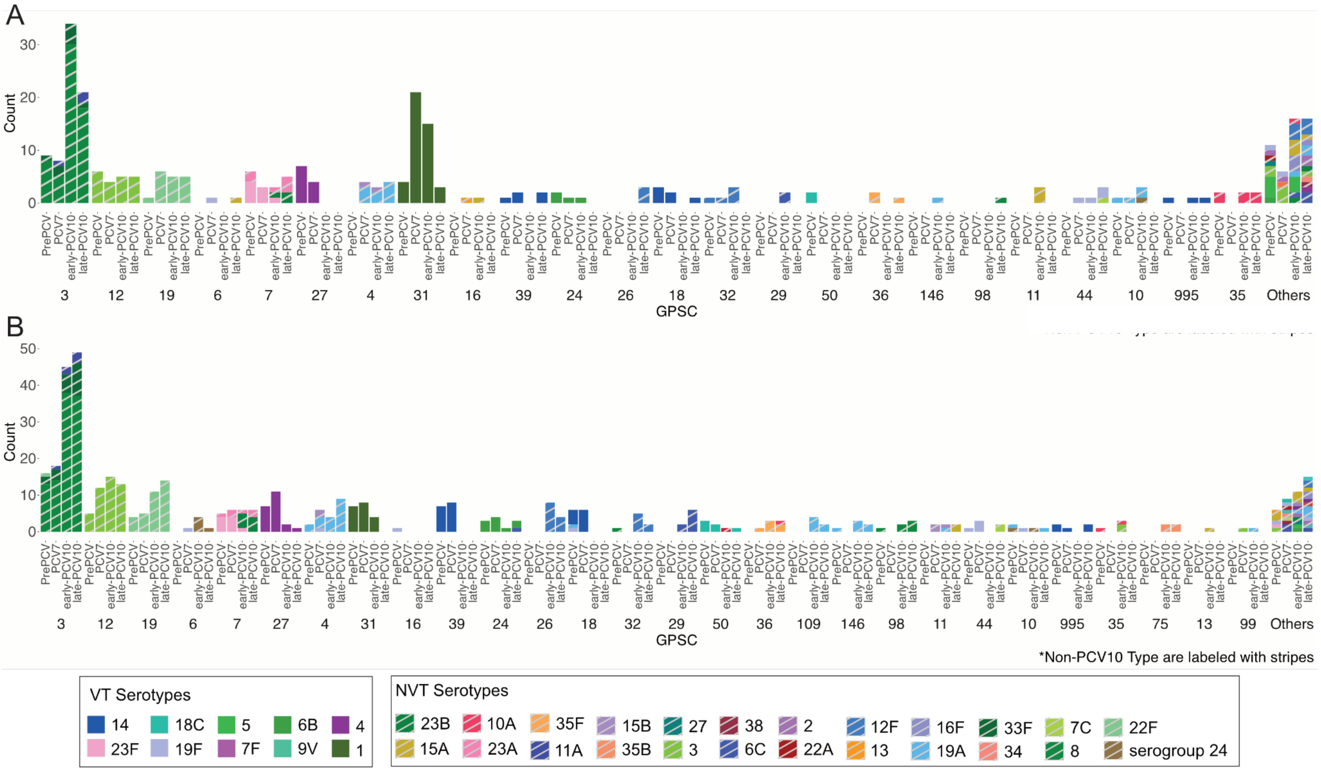

Figure S7 - Distribution of GPSCs across vaccine periods in the adult aged 18-64 years old (A) and aged ≥65 years old (B), coloured by serotype composition. NVTs are shown with stripes. Minor GPSCs are grouped into “Others”.

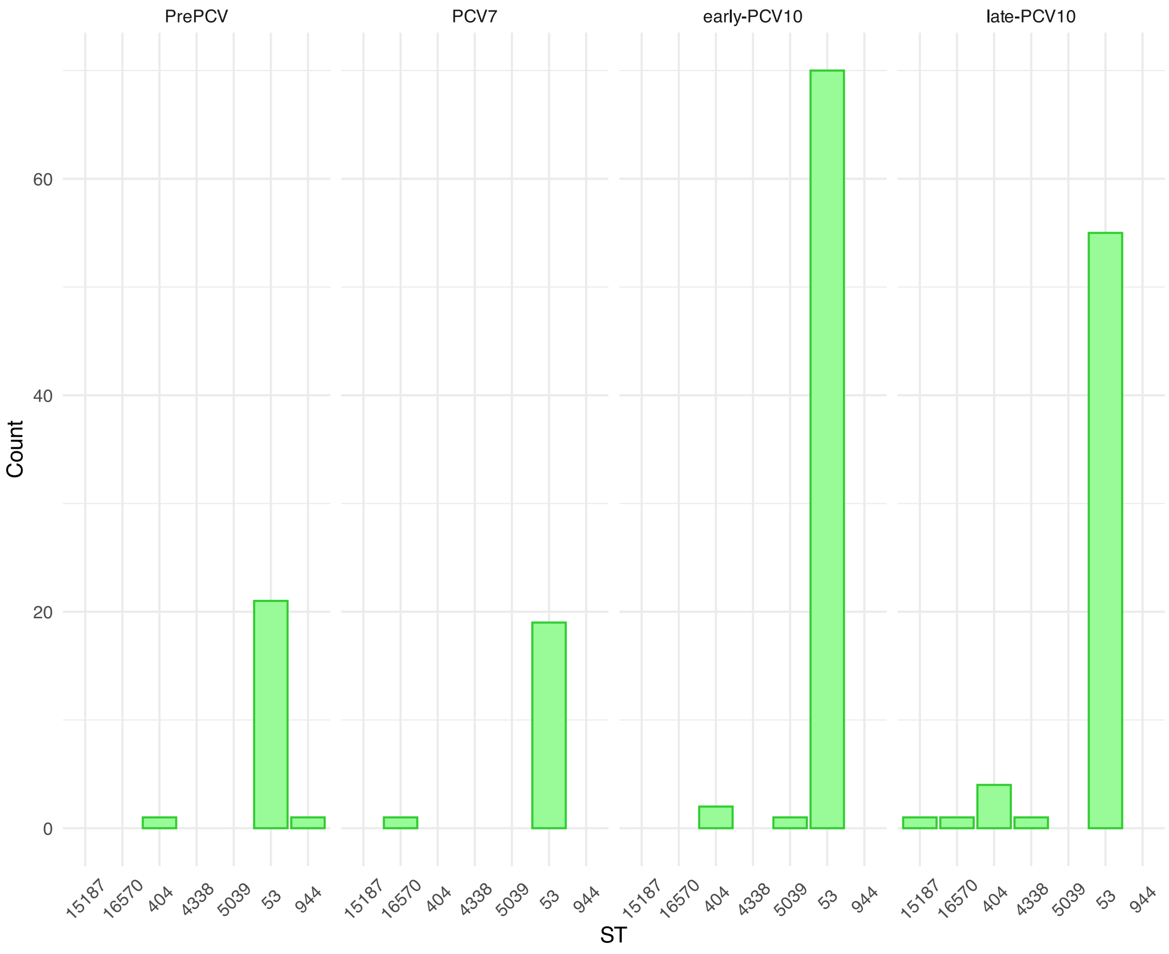

Figure S8 - STs driving the increase in serotype 8. ST53 is the main driver, and is always the dominant ST expressing serotype 8. However, the increase is not statistically significant.

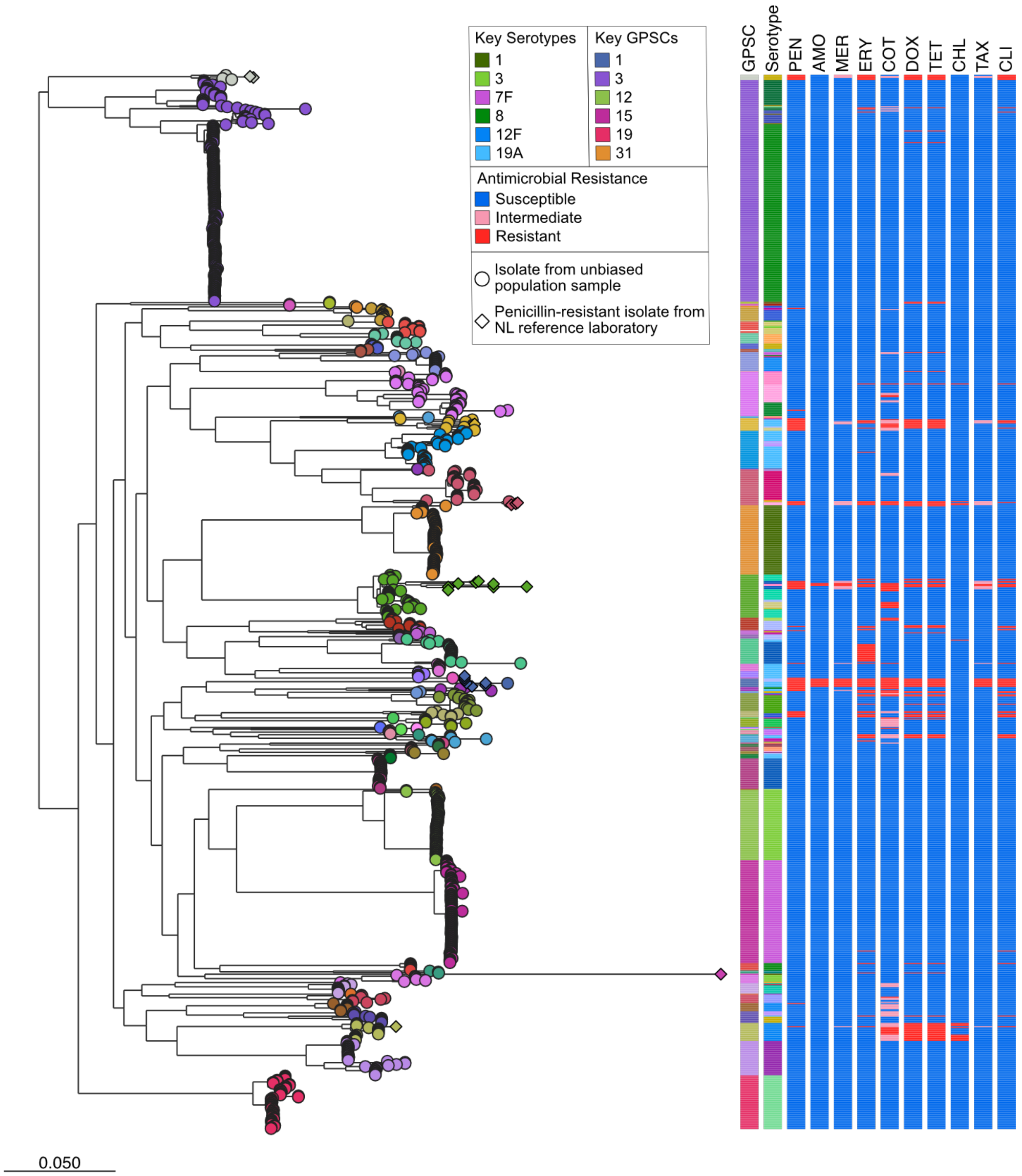

Figure S9 - A maximum-likelihood phylogeny of the S. pneumoniae (n = 1,004) study population. Isolates are coloured according to GPSC lineages. The predicted antimicrobial susceptibility was based on the genome data and interpreted according to the Clinical and Laboratory Standards Institute (CLSI). Meningitis cutoff was used to interpret the MIC values of penicillin (≥0.12µg/ml as resistant), cefotaxime (≥2 µg/ml as resistant) and ceftriaxone (≥2 µg/ml as resistant). Nodes are coloured by GPSC. A pseudo-genome alignment was generated by mapping reads to the S.pneumoniae reference genome (ATCC_700669) using SMALT (v0.7.4). SNPs were identified with snp-sites (v2.5.1) from the pseudo-genome, and a maximum-likelihood phylogenetic tree was built with FastTree (v2.1.0)

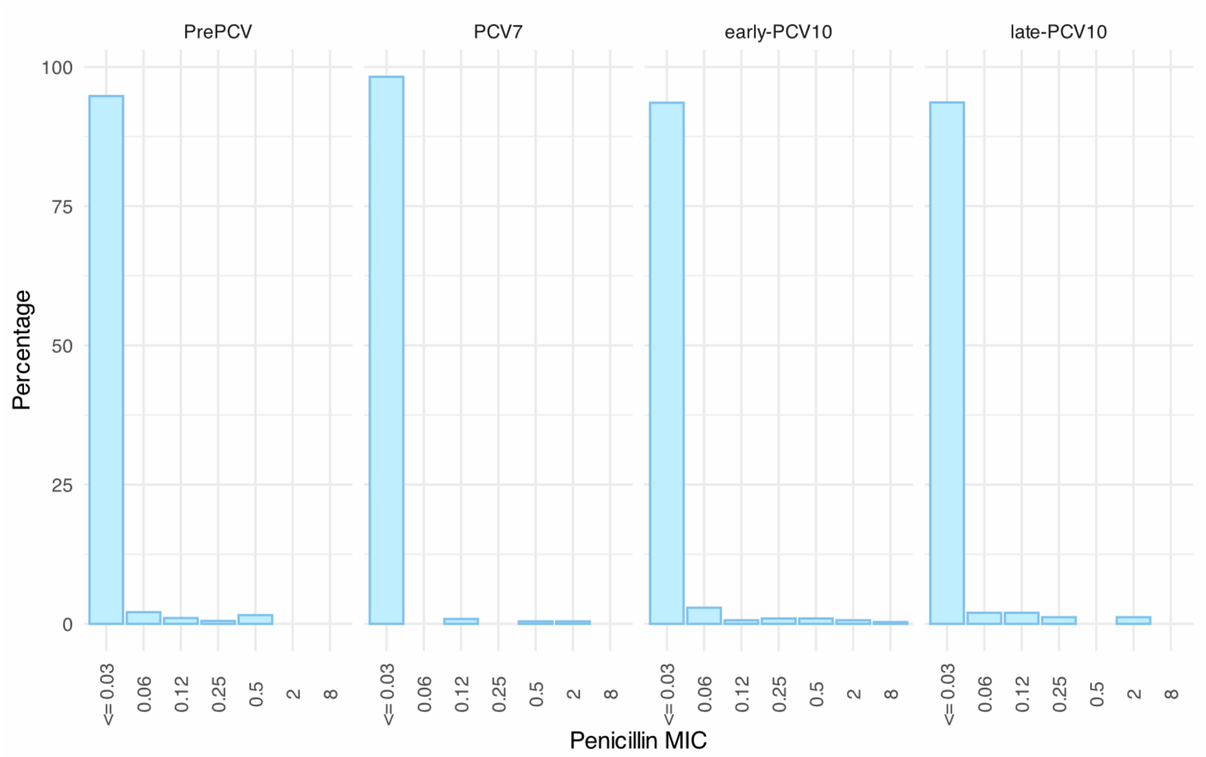

Figure S10 - Changes in predicted MIC based on genotype for penicillin in the isolates collected in the unbiased population sample over the course of this study. Most isolates have a in silico predicted MIC <= 0.03, and this does not significantly change over the course of the study (p = 0.14).

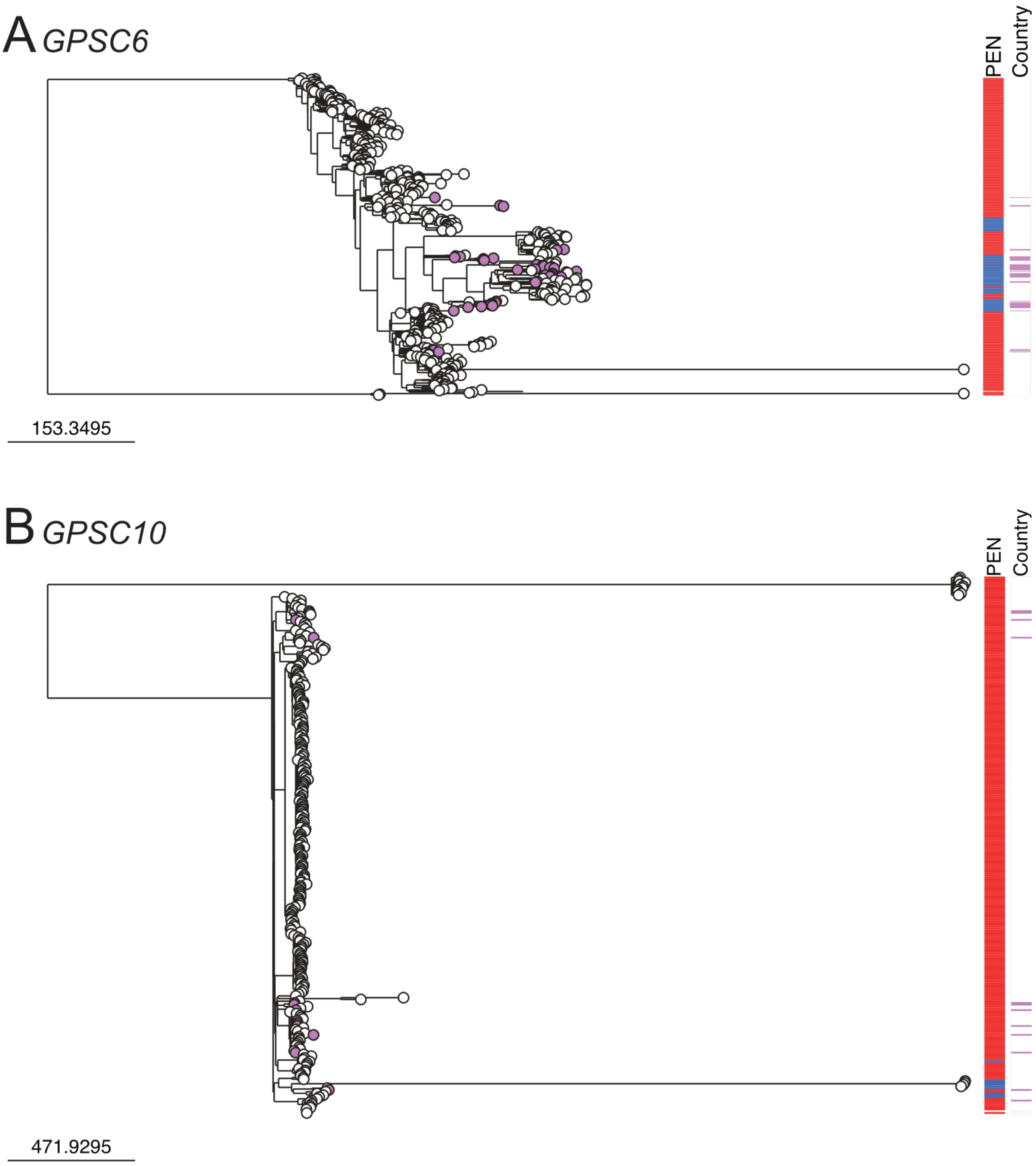

Figure S11 - Recombination-free phylogeny of GPSC6 and GPSC10 S. pneumoniae showing penicillin resistance (Red = resistant, blue = Susceptible). Samples from the Netherlands (purple) do not cluster together, instead being interspersed with other countries (white). The recombination-free phylogeny was constructed for each GPSC using Gubbins (v3.2.1), with alignments generated by mapping reads to lineage-specific reference genomes. A total of 434 GPSC6 and 302 GPSC10 S. pneumoniae genomes were used for this analysis, with the genomes of isolates from outside the Netherlands dataset being provided by the GPS1 dataset.

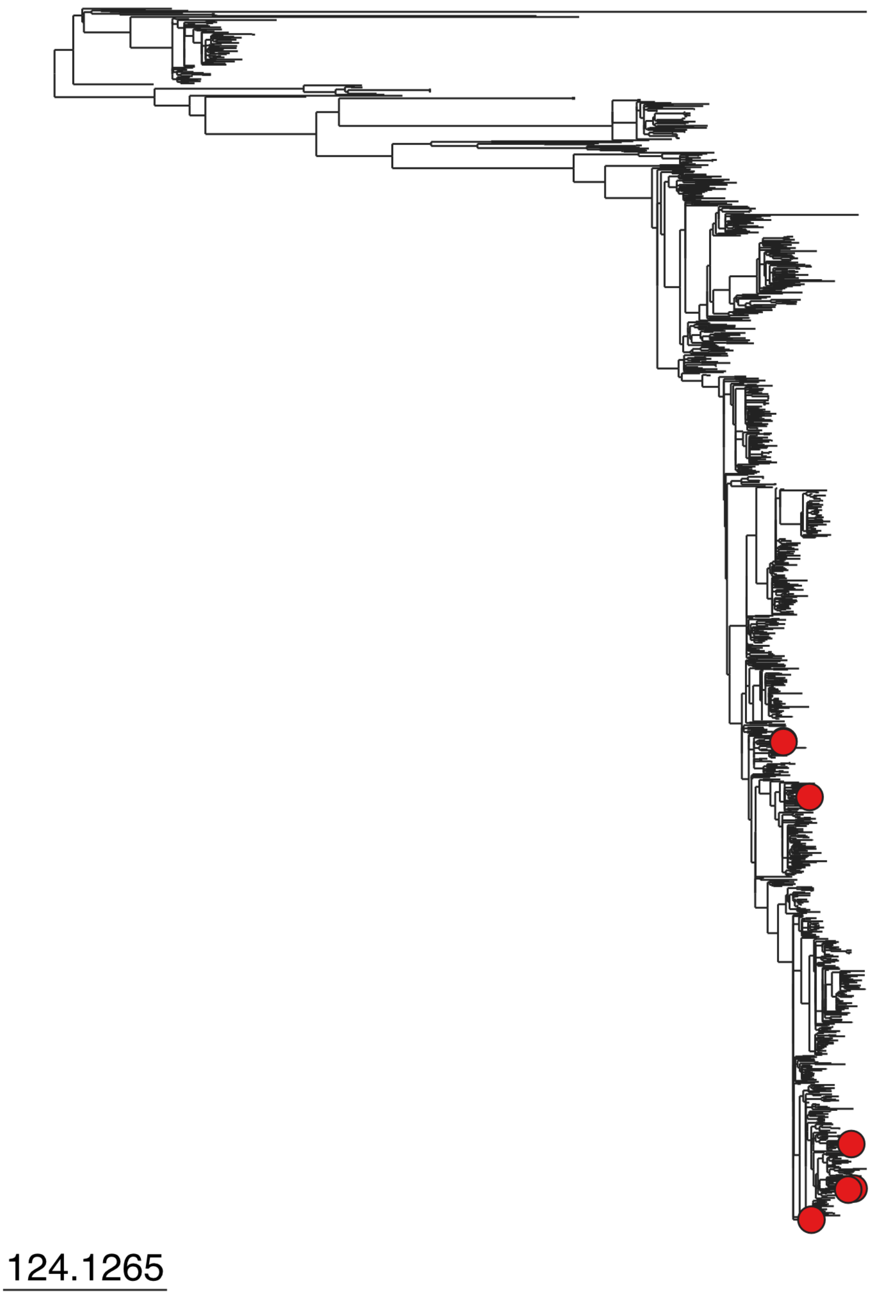

Figure S12 - Recombination-free phylogeny of 1173 S. pneumoniae GPSC1 isolates, using samples from the GPS1 dataset alongside the bacteraemia dataset. Isolates from the Netherlands are shown with red. As they do not form a single monophyletic clade, multidrug-resistant GPSC1 was likely introduced to the Netherlands multiple times. Gubbins (v3.2.1) was used to construct this phylogeny based on a SNP alignment generated by SKA2. All eight GPSC1 S. pneumoniae isolates from this study were included in the phylogeny.

Table S1 - Count and percentage of isolates that are resistant/intermediate and susceptible to antibiotics per age group across the different vaccine periods. *The number and percentage of isolates resistant to each antimicrobial is indicated. [a] The predicted antimicrobial susceptibility are predicted from genome data and interpreted according to Clinical and Laboratory Standards Institute (CLSI, M100-ED28:2018 [18]). Meningitis clinical breakpoints were used to interpret the MIC values of penicillin (≥0.12μg/ml as resistant), cefotaxime (≥2 μg/ml as resistant) and ceftriaxone (≥2 μg/ml as resistant), which are identical between CLSI and EUCAST.*

<18 years old (n = 48; 100%)										
Vaccine Period	*Antibiotic*	*Penicillin[a]*	*Amoxicillin*	*Meropenem*	*Cefotaxime[a]*	*Erythromycin*	*Clindamycin*	*Chloramphenicol*	*Cotrimoxazole*	*Doxycycline*
Pre-PCV	*Resistant/Intermediate*	-	-	-	-	3 (30%)	1 (10%)	-	1 (10%)	1 (10%)
	Susceptible	10 (100%)	10 (100%)	10 (100%)	10 (100%)	7 (70%)	9 (90%)	10 (100%)	9 (90%)	9 (90%)
PCV7	*Resistant/Intermediate*	-	-	-	-	4 (44%)	2 (22%)	-	3 (33%)	2 (22%)
	Susceptible	9 (100%)	9 (100%)	9 (100%)	9 (100%)	5 (56%)	7 (78%)	9 (100%)	6 (67%)	7 (78%)
		-	-	-	-	-	-	-	2 (14%)	-

early-PCV10	*Resistant/Intermediate*									
	Susceptible	*14 (100%)*	*14 (100%)*	*14 (100%)*	*14 (100%)*	*14 (100%)*	*14 (100%)*	*14 (100%)*	*12 (86%)*	*14 (100%)*
late-PCV10	*Resistant/Intermediate*	*1 (7%)*	*-*	*-*	*-*	*-*	*-*	*-*	*1 (7%)*	*1 (7%)*
	Susceptible	*14 (93%)*	*15 (100%)*	*15 (100%)*	*15 (100%)*	*15 (100%)*	*15 (100%)*	*15 (100%*	*14 (93%)*	*14 (93%)*
18-64 years old (n = 368; 100%)										
Pre-PCV	*Resistant/Intermediate*	*3 (4%)*	*-*	*-*	*-*	*4 (5%)*	*1 (1%)*	*-*	*9 (12%)*	*3 (4%)*
	Susceptible	*71 (96%)*	*74 (100%)*	*74 (100%)*	*74 (100%)*	*70 (95%)*	*73 (99%)*	*74 (100%)*	*65 (88%)*	*71 (96%)*
	Resistant/Intermediate	*3 (3%)*	*-*	*-*	*1 (1%)*	*4 (5%)*	*2 (2%)*	*-*	*5 (6%)*	*3 (3%)*

PCV7										
	Susceptible	85 (97%)	88 (100%)	88 (100%)	87 (99%)	84 (96%)	86 (98%)	88 (100%)	83 (94%)	85 (97%)
early-PCV10	Resistant/Intermediate	7 (6%)	1 (1%)	3 (2.4%)	3 (2%)	5 (4%)	4 (3%)	1 (1%)	11 (9%)	9 (7%)
	Susceptible	118 (94%)	124 (99%)	122 (98%)	122 (98%)	120 (96%)	121 (97%)	124 (99%)	114 (91%)	116 (93%)
late-PCV10	Resistant/Intermediate	6 (7%)	-	2 (3%)	1 (1%)	75 (93%)	6 (7%)	1 (1%)	12 (15%)	8 (10%)
	Susceptible	75 (93%)	81 (100%)	79 (98%)	80 (99%)	6 (7%)	75 (93%)	80 (99%)	69 (85%)	73 (90%)
≥65 years old (n = 563; 100%)										
Pre-PCV	Resistant/Intermediate	3 (3%)	-	-	-	10 (9%)	5 (5%)	1 (1%)	8 (8%)	5 (5%)
	Susceptible	104 (97%)	107 (100%)	107 (100%)	107 (100%)	97 (91%)	102 (95%)	106 (99%)	99 (93%)	102 (95%)

PCV7	Resistant/Intermediate	1 (1%)	-	-	-	10 (8%)	4 (3%)	1 (1%)	8 (6%)	6 (5%)
	Susceptible	128 (99%)	129 (100%)	129 (100%)	129 (100%)	119 (92%)	125 (97%)	128 (99%)	121 (94%)	123 (95%)
early-PCV10	Resistant/Intermediate	4 (2%)	-	-	-	6 (4%)	5 (3%)	5 (3%)	24 (14%)	14 (8%)
	Susceptible	168 (98%)	172 (100%)	172 (100%)	172 (100%)	166 (97%)	167 (97%)	167 (97%)	148 (86%)	158 (92%)
late-PCV10	Resistant/Intermediate	4 (3%)	-	1 (1%)	1 (1%)	3 (2%)	3 (2%)	4 (3%)	15 (10%)	9 (6%)
	Susceptible	151 (97%)	155 (100%)	154 (99%)	154 (99%)	152 (98%)	152 (98%)	151 (97%)	140 (90%)	146 (94%)

Table S2 – The proportion of multidrug-resistance in 16 pneumococcal lineages detected in the Gelderland area, the Netherlands, 2000-2020. [a] Isolates were considered MDR isolates if these shown to be intermediate or resistant to three classes of antimicrobials

GPSC	*Number of MDR isolates*[a]	*Number of isolates*	*Percentage of MDR isolates in GPSC*
1	8	8	100%
5	1	4	25%
6	5	41	12%
7	1	42	2%
9	5	5	100%
10	9	12	75%
11	1	11	9%
16	4	34	12%
18	1	24	4%
24	2	17	12%
26	11	17	65%
43	1	1	100%
44	1	12	8%
47	5	6	83%
99	3	7	43%
101	2	2	100%

[a]Number of pneumococcal isolates that are non-susceptible to at least three classes of antibiotics. The antimicrobial susceptibilities are predicted by the presence and absence of the known resistance genes/mutations using genome data.
